# Pre-Exercise Caffeine and Sodium Bicarbonate: Their Effects on Isometric Mid-Thigh Pull Performance in a Crossover, Double-Blind, Placebo-Controlled Study

**DOI:** 10.3390/sports12080206

**Published:** 2024-07-29

**Authors:** Celil Kaçoğlu, İzzet Kirkaya, Halil İbrahim Ceylan, Gilmara Gomes de Assis, Paulo Almeida-Neto, Serdar Bayrakdaroğlu, César Chaves Oliveira, Ali Özkan, Pantelis T. Nikolaidis

**Affiliations:** 1 Department of Coaching Education, Faculty of Sport Sciences, Eskişehir Technical University, Eskişehir 26555, Türkiye; ckacoglu@eskisehir.edu.tr; 2 Department of Coaching Education, Faculty of Sport Sciences, Yozgat Bozok University, Yozgat 66100, Türkiye; izzet.kirkaya@yobu.edu.tr (İ.K.); ali.ozkan@yobu.edu.tr (A.Ö.); 3 Department of Physical Education of Sports Teaching, Faculty of Sports Sciences, Atatürk University, Erzurum 25240, Türkiye; halil.ibrahimceylan60@gmail.com; 4 Araraquara School of Dentistry, São Paulo State University (UNESP), Araraquara 01049-010, Brazil; dr.deassisgg@gmail.com; 5 Department of Physical Education, Federal University of Rio Grande do Norte, CCS-UFNR, Natal 59078-900, Brazil; paulo.neto.095@ufrn.edu.br; 6 Department of Coaching Education, Movement and Training Sciences, School of Education and Sport, Gumushane University, Gumushane 29100, Türkiye; bayrakdaroglu85@gmail.com; 7 Polytechnic Institute of Viana do Castelo, School of Sports and Leisure, 4960-320 Viana do Castelo, Portugal; cesarchaves@esdl.ipvc.pt; 8 School of Health and Caring Sciences, University of West Attica, 11521 Athens, Greece

**Keywords:** caffeine, sodium bicarbonate, isometric mid-thigh pull, supplements, ergogenic aids, combined effect, strength

## Abstract

Caffeine and sodium bicarbonate are extensively researched ergogenic aids known for their potential to enhance exercise performance. The stimulant properties of caffeine on the central nervous system, coupled with the buffering capacity of sodium bicarbonate, have been associated with improved athletic performance. This has led to investigations of their combined effects on strength. The aim of the present study is to investigate the effect of isolated and combined caffeine and sodium bicarbonate consumption on strength using the isometric mid-thigh pull test (IMTP). Nineteen male college students (age 23.6 ± 1.6 years) participated in this crossover, double-blind, placebo-controlled study. They were exposed to the following conditions: control (no supplement), placebo (20 g maltodextrin), caffeine (6 mg/kg), sodium bicarbonate (0.3 g/kg), and a combination of caffeine and sodium bicarbonate. Supplements and placebo were diluted in water and consumed 60 min prior to the IMTP tests. Two 5 s IMTP trials were performed at 40–60% and 60–80% of One Repetition of Maximum (1RM) with a 60 s rest between. Consumption of caffeine or Caf + NaHCO_3_ did not significantly change peak IMTP strength values at any intensity (*p* = 0.110). The peak IMTP values did not show significant differences between conditions or from control condition values (1091 ± 100 N) to Caf (1224 ± 92 N), NaHCO_3_ (1222 ± 74 N), and Caf ± NaHCO_3_ (1152 ± 109 N). However, the test of the results of the ANOVA analysis of repeated measures of effect within the caffeine condition was significant for the increase in IMTP relative strength compared to control (*p* < 0.05). Thus, the IMTP force values increased significantly from control to Caf (*p* = 0.016) and from Pla to Caf (*p* = 0.008), but not for other comparisons (*p* > 0.05). In summary, caffeine supplementation alone, taken 60 min before exercise, positively affects submaximal strength performance. In contrast, sodium bicarbonate, whether taken alone or in combination with caffeine, does not enhance submaximal strength in the IMTP tests.

## 1. Introduction

Winning sports competitions requires effective work in various domains, including training methods and nutritional supplements [1,2]. With only a 1% performance difference between medalists and non-medalists in the 2012 and 2016 Olympics, even slight improvements from appropriate supplementation can significantly improve performance. Therefore, supplementation is crucial to the preparation of elite athletes [2]. Caffeine, valued for its stimulant properties, is consumed by 74% of elite athletes as an ergogenic aid before and during physical activity or competition [3]. Its easy access, low cost, and minimal adverse effects contribute to its global acceptance [4]. Studies show that low to medium doses (1–6 mg/kg) of caffeine enhance sports performance by increasing intracellular calcium and free fatty acid mobilization and antagonizing adenosine receptors, which benefits endurance performances [5].

Research also indicates small ergogenic effects on power in isokinetic, isometric, and 1RM strength tasks, with effect sizes of 0.16, 0.19, and 0.20, respectively [6,7,8,9]. While caffeine benefits aerobic exercises, its effects on anaerobic performance are less certain. However, sodium bicarbonate is known for its performance-enhancing effects on high-intensity tasks such as sprinting, swimming, boxing, rowing, and muscular endurance. Taken at 0.3 g/kg of body weight 60 to 120 min before exercise, it increases blood bicarbonate concentration, helping maintain pH balance by buffering hydrogen ions [10,11]. This reduces metabolic acidosis, delaying fatigue [12]. Sodium in the bicarbonate also helps neutralize acidity and increase plasma volume, improving anaerobic activity performance by diluting H+ concentrations [11,13].

A meta-analysis found that caffeine and sodium bicarbonate similarly improve Yo-Yo test performance [14]. While both are ergogenic individually for various exercises, studies on their combined effects are limited, particularly on acute strength outcomes [15,16,17]. Previous research has primarily focused on the benefits of each supplement in isolation, leaving a significant gap in understanding their potential synergistic effects. This is particularly important for strength performance, where acute enhancements can be critical.

Mechanistically, caffeine acts as a central nervous system stimulant by blocking adenosine receptors, particularly A1 and A2A, which leads to an increase in the release of neurotransmitters such as dopamine, norepinephrine, and acetylcholine. This increase enhances motor unit recruitment and overall force production, thus improving neuromuscular activation and performance during exercise [18,19]. Additionally, caffeine’s effect on calcium ion (Ca^2+^) mobilization within muscle cells facilitates greater force production by each motor unit, potentially delaying fatigue and enhancing muscle contraction strength [19,20]. Sodium bicarbonate, on the other hand, is effective in buffering lactic acid produced during high-intensity exercise. When ingested, it dissociates into sodium and bicarbonate ions, which help neutralize the hydrogen ions (H^+^) that accumulate during anaerobic metabolism. This buffering action helps maintain pH balance in the blood and muscle tissues, delaying the onset of fatigue and allowing for sustained high-intensity performance. This is particularly beneficial for activities that require repeated bursts of power and speed [21,22,23]. The combination of these effects could theoretically result in a synergistic enhancement of strength performance by improving neuromuscular activation and delaying fatigue.

Existing studies on the combined effects of caffeine and sodium bicarbonate have shown mixed results. For instance, a comprehensive review by Grgic, (2021) critically examined the existing literature on the combined effects of caffeine and sodium bicarbonate. The review highlighted mixed results, with some studies showing synergistic effects and others not, emphasizing the need for further research to clarify these interactions under different conditions and types of exercise [24]. Additionally, another study by Kilding et al., (2012) showed that caffeine and sodium bicarbonate enhanced performance individually. Combined, the supplements did not produce an additive effect, suggesting that while they are effective on their own, their combined use may not provide additional benefits [25]. Lastly, a recent randomized controlled trial study found that co-ingestion of caffeine and sodium bicarbonate did not result in significantly greater improvements in repeated-sprint performance compared to each supplement taken alone. This suggests that while both supplements are effective individually, their combined use may not provide additional benefits [26]. Regarding the studies, these discrepancies suggest that the combined ergogenic effects may be context-dependent, varying with the type of exercise and the specific conditions of the study.

Considering the information and studies mentioned above, our study aims to address this gap by systematically investigating the combined effects of caffeine and sodium bicarbonate on acute strength performance using the isometric mid-thigh pull test (IMTP). The IMTP test was utilized in this study due to its proven efficacy in evaluating maximal strength and rate of force development, which are critical components of athletic performance. The IMTP test is a standardized measure that provides reliable and valid assessments of an athlete’s strength, making it a pertinent choice for studies examining the impact of ergogenic aids. Its application in both research and practical settings further underscores its utility in detecting performance changes following nutritional or training interventions. By examining the impact of this combination on IMTP performance, we aim to offer new insights into the synergistic potential of these supplements in improving strength performance. This may provide a new strategy for athletes looking to maximize their competitive advantage. Given the well-established ergogenic properties of caffeine and sodium bicarbonate, we hypothesize that the combined supplementation will result in significant improvements in strength performance, as measured by the IMTP, compared to the isolated use of each supplement. The present study aimed to investigate the effect of isolated and combined consumption of caffeine and sodium bicarbonate on strength using IMTP.

## 2. Materials and Methods

This study was carried out in the Laboratory of Human Athletic Performance of the Faculty of Sport Sciences of Eskişehir Technical University. The participants were assigned to a crossover, double-blind, placebo-controlled trial. They were verbally informed about the purpose and methods and the ergogenic effects of caffeine and sodium bicarbonate consumption on performance and that they would be blind to supplementation or placebo conditions before they signed a written informed consent form according to the guidelines of the Declaration of Helsinki and approved by the Ethics Committee of the Eskişehir Technical University (approval number: 13884, 30 June 2020). Also, our study was registered with ClinicalTrials.gov with NCT05883046.

### 2.1. Participants

Nineteen male students from the Sport Sciences Faculty who were physically active and healthy did not use ergogenic supplements in the last 3 months and did not consume high doses of caffeine (≤120–170 mg/d) were included in this study. The sample size was determined a priori by power analysis using G*Power^®^ (Version 3.1.9.7, Brunsbuttel, Germany). The alpha (α) value was determined as 0.05, the effect size was 0.3, the power value (1-β) was 0.90 for the repeated measures statistical test (within factor) statistical test, and the minimum number of participants resulted in 19 (power: 0.91, critical F (4.0): 2.49). The characteristics of the participants are shown in Table 1.

Participants were informed verbally and in writing that they needed to refrain from consuming any form of caffeine (including coffee, tea, energy drinks, cocoa, chocolate, or caffeine-containing medications) for at least 24 h before the intervention. Every time they visited the laboratory, they were asked and checked to ensure that they had adhered to this requirement by abstaining from these food and drink items.

### 2.2. Study Design

All participants visited the laboratory one week before the measurements for familiarization, and the tests were applied individually so that the participants were allowed to experiment with the evaluation procedures. Participants made six visits to the laboratory in total. The first visit was for familiarization, and the subsequent five visits were for the experimental conditions: control (no supplement), placebo (maltodextrin), caffeine, sodium bicarbonate, and caffeine combined with sodium bicarbonate. The order of these conditions was randomized and double-blind, and the researcher who conducted the measurements was unaware of the specific condition during each visit. Participants’ body weight was measured using a medical digital weighing scale (Seca, Vogel & Halke, Hamburg, Germany), while their height was assessed with a stadiometer (Holtain Ltd., Crosswell, Crymych, UK). Additionally, their body fat percentage was determined using a four-compartment bioelectric impedance analyzer (Tanita MC 180MA, Tanita, Tokyo, Japan). It was also measured on the day of familiarization, and the isometric mid-thigh pull (IMTP) tests were applied at an intensity close to the perceived maximal effort. During the isometric mid-thigh pull (IMTP) test, participants were asked to rate their perceived exertion using Borg’s CR-10 scale. They were prompted with the standard question, “How hard do you feel your leg muscles are working?”. Participants had access to the complete scale throughout the test [27]. A wash-out period of at least 7 days was given between measurements, and all measurements were made on the same day of the week and at the same time of day (11:00–14:00) to avoid the time-of-day confounders. Participants were asked not to participate in heavy physical activities during the whole intervention time and not to consume additional supplements except those used in this study. During the investigation, control condition measurements were also taken to observe the possible effects of daily physical activity.

### 2.3. Isometric Mid-Thigh Pull Evaluations (IMTP)

There are various tests of maximum strength, one of which is the isometric mid-thigh pull test, which has gained significant popularity in recent years. Recent studies have demonstrated that load cell devices are valid and reliable tools for assessing the IMTP test. Montoro-Bombu et al., (2023) found that a load cell sensor-based device showed excellent validity and reliability, with Intraclass Correlation Coefficients (ICCs) ranging from 0.92 to 0.98 and coefficients of variation (CVs) below 5%, indicating consistent measurements of isometric strength [28]. Similarly, a systematic review with meta-analysis by Grgic et al., (2020) further supported these findings, with ICC values typically above 0.90 across multiple studies, confirming the IMTP test’s reliability for assessing maximum isometric strength [29]. A strain gauge was placed in the middle of a chain fixed to the bar, and a platform on the ground was used for the measurements (PCE-FG-1K, PCE Instruments, Meschede, Germany). In the absence of force platforms, strain gauges serve for strength measurements, although they may register values 8–10% lower [30,31]. During the test, no tension should be applied to the bar during joint angle measurements, and the knee and hip joints should form an angle of approximately 145 degrees. The participant’s body should be in an upright position (without transferring their body weight backward), with feet hip-width parallel. They would grip the middle of the bar, while the bar should be placed in the middle of the calf (inguinal area). Participants were explained that they should place the bar aligned with the crease area (gluteal crease) and that their hands should be positioned aligned with the outer parts of their thighs. Three repetitions of submaximal attempts (meaning 50%, 75%, and 90% of perceived maximal effort, respectively) were made for 5 s. The tests occurred approximately 48 h after familiarization.

In the measurement sessions, a standard warm-up was performed that involved 3 min of cycling and clean-like exercises (including 10 bodyweight squats, 10 bodyweight lunge walks, and 10 glute bridges). About three minutes after standard warm-up, two submaximal IMTP trials were performed at increasing intensities for 5 s (approximately 40–60% and 60–80% of their individually perceived 1RM, respectively, with 60 s rest intervals). After a reasonable passive rest interval (approximately 2–3 min) was administered after the warm-up period, participants assumed the correct position for the measurement, the joint angles were determined without applying tension, and the height of the bar was determined individually for each participant by adjusting the length of the fixed bar chain by these angles. The researcher used external cues, like “push your feet into the ground as fast and hard as possible”, as the standard explanation before the test, emphasizing maximal force production.

**Figure 1:**
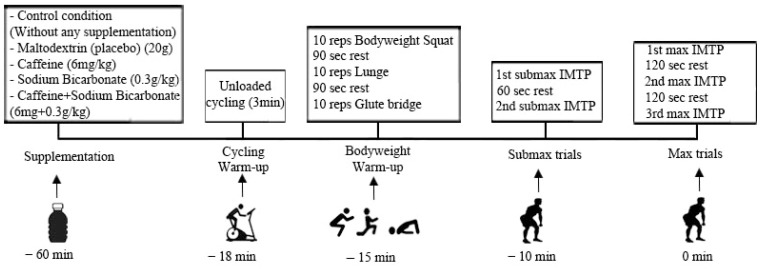
Flowchart of the experimental design of the study.

After a few moments of standing as still as possible, the participants were given a countdown of “3, 2, 1, GO” to ensure that they were ready to give maximal effort on each attempt. During the test, the researcher strongly repeated the standard verbal encouragement statements “push, harder”. Three repetitions were performed at 2 min intervals. During the test, a graphical curve formation was observed on the computer screen. The flow chart of the experimental steps for each test condition is represented in Figure 1. Data were checked in the 2 min between measurements, and if there was a difference of more than 250 N between measurements, if the tension in the chain disappeared because the participant applied a preliminary downward movement to the bar before the measurement, or if the participant turned his body weight backward, the measurement was repeated. Data obtained from repetitions that reached maximum (N) and relative force values (N/Kg) were saved to an Excel^®^ sheet for the statistical analysis [32,33,34].

### 2.4. Supplementation Protocols

The preparation, provision, and management of the blind conditions for participants and investigators was carried out by a researcher who did not participate in the strength measurements. After the familiarization process, the subjects went through 5 crossover conditions: Con, Pla, Caf, NaHCO_3_, and Caf + NaHCO_3_. Mixtures for Pla, Caf, NaHCO_3_, and Caf + NaHCO_3_ were prepared by mixing and dissolving them with 200 mL of water in black-coated pet bottles that were subsequently delivered to another researcher, who then identified the received mixtures with a code number. Under Pla conditions, participants consumed 20 g of maltodextrin (Alfasol^®^, Kimbiotek, Türkiye). Maltodextrin was chosen as the placebo due to its properties as an inert substance with minimal physiological impact at the dose used; under Caf conditions, participants consumed 6 mg/kg of caffeine obtained from dosing the content of 200 mg capsules (Nature’s Supreme^®^ Caffeine, İstanbul, Türkiye) [9,35,36]; in the NaHCO_3_ condition, participants consumed 0.3 g/kg of sodium bicarbonate (Alfasol^®^, Kimbiotek, Türkiye) dissolved in water; and finally, under Caf + NaHCO_3_ condition, participants consumed 6 mg/kg of caffeine and 0.3 g/kg of sodium bicarbonate per kilogram of body weight dissolved together in water. All supplements were consumed 60 min before the tests to reach maximum blood concentrations at the evaluation time. Immediately after consumption, both the Con and intervention participants rested in a quiet laboratory environment (Eskişehir Technical University, Laboratory of Human Athletic Performance) until the IMTP tests [37,38].

### 2.5. Statistical Analysis

A one-way repeated measures ANOVA was performed to determine whether there were statistically significant differences in IMTP values between conditions. All analyses were performed using the SPSS (v20.0) statistical package program. For all analyses, a significance of *p* < 0.05 was considered. Data were provided as mean and standard deviation. There were no outliers, and the data were normally distributed at each time point, as assessed by the box plot and Shapiro–Wilk test (*p* > 0.05). The assumption of sphericity for peak values was not met, as assessed by Mauchly’s sphericity test, X^2^(9) = 22.710, *p* = 0.07. Therefore, a Greenhouse–Geisser correction was applied, and the epsilon (ε) was 0.625, calculated according to Greenhouse and Geisser [39], and was used to correct the one-way repeated measures ANOVA. Effect size measurements were analyzed using Eta-square (η^2^), being interpreted by magnitude [40]: small < 0.20; average > 0.20 and <0.50; large > 0.50. The delta variation (Δ%) of the control condition was calculated by the formula: Δ% = [(Supplementation/Control) − 1) × 100]. In this way, the Δ% values were compared with each other using the student-dependent “T” test. The effect size between the differences was verified by Cohen’s d test, being interpreted by the magnitude: small < 0.20; medium > 0.20 and <0.50; large > 0.50. We performed post hoc sample power analyses using G*Power software (Version 3.1.9.7, Brunsbuttel, Germany).

## 3. Results

The isolated and combined consumption of caffeine and sodium bicarbonate did not cause statistically significant changes in the peak IMTP strength values under any condition F (2.213, 44.997). IMTP peak values did not differ between conditions besides a non-statistical increase from control condition values (1091 ± 100 N) to Caf (1224 ± 92 N), NaHCO_3_ (1222 ± 74 N), and Caf ± NaHCO_3_ (1152 ± 109 N).

When making comparisons for the relative values (N/Kg) of IMTP strength, we identified that there was an increase in the control intervention for Caf (0.002 (95% CI, 0.004 to 0.000) N/kg, *p* =0.016) and from Pla to Caf (0.002 (95% CI, 0.003 to 0.000) N/kg, *p* = 0.008), but not in the other pairs (*p* > 0.05) (Table 2).

**Figure 2:**
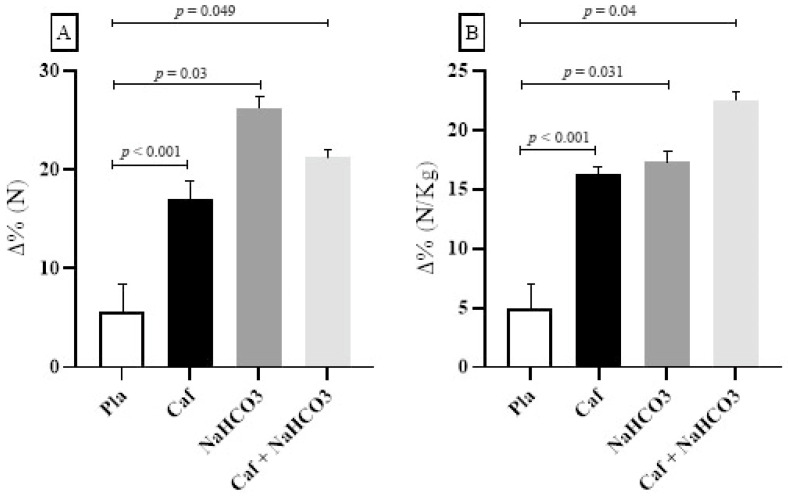
Comparisons of delta variation values (Δ%) in relation to the control condition. (**A**): Δ% peak in Newtons. (**B**): Δ% Newtons/kilogram. (N): Newtons. (N/Kg): Newtons per kilogram. Pla: placebo. Caf: caffeine. NaHCO_3_: sodium bicarbonate. Caf + NaHCO_3_: caffeine + sodium bicarbonate.

The comparisons in Figure 2 indicated that there was a significant difference between (i) the Δ% variation of the Pla and Caf groups ([N]: effect size: 0.954, power: 0.970; [N/kg]: effect size: 0.956, power: 0.970) (Figure 2A,B), (ii) the Δ% variation of the Pla and NaHCO_3_ groups ([N]: effect size: 0.501, power: 0.670; [N/kg]: effect size: 0.542, power: 0.730), and (iii) the Δ% variation of the Pla and Caf + NaHCO_3_ groups ([N]: effect size: 0.315, power: 0.370; [N/kg]: effect size: 0.507, power: 0.680).

There were no significant differences between the Δ% variation: (i) Caf versus NaHCO_3_ ([N]: *p* = 0.067. [N/Kg]: *p* = 0.3), (ii) Caf versus Caf + NaHCO_3_ ([N]: *p* = 0.7. [N/Kg]: *p* = 0.5), and NaHCO_3_ versus Caf + NaCO_3_ ([N]: *p* = 0.2. [N/Kg]: *p* = 0.2).

## 4. Discussion

This study aimed to investigate the effects of isolated caffeine, isolated sodium bicarbonate, combined caffeine–sodium bicarbonate, and placebo supplementation on the isometric performance of mid-thigh pull. Our study’s findings indicate that caffeine alone positively affects submaximal strength performance, whereas sodium bicarbonate, whether isolated or combined with caffeine, does not enhance strength performance.

Consistent with our findings, a meta-analysis performed by Grgic et al. [14] on the effects of sodium bicarbonate consumption on muscular strength and endurance. It was reported that sodium bicarbonate does not exert ergogenic effects on muscle strength. Burke et al. [41] investigated the effects of 6 mg/kg of body mass caffeine supplementation on IMTP performance and, as a result, concluded that maximal isometric strength did not increase with caffeine consumption. Different researchers have examined the effect of caffeine on strength using various methods.

Alkatan et al. [42] study used 5 mg/kg ingested 45 min before exercise for male weightlifters and observed a 2.2% increase in IMTP performance. Behrens et al. [43] used the same amount, ingested 60 min before exercise, on physically active university students (female and male) and observed an increase of 14.3% in their isokinetic plantar flexion. Alternatively, the study by Arazi et al. [44], which examined the effect of caffeine on 1RM leg press performance in male athletes, did not obtain significant results. Finally, the size of caffeine effects on maximal strength performance of approximately 0.20, according to meta-analysis studies, was parallel to the findings of our study (0.62) [7,45].

Previously, it was pointed out that the effect of the combination of caffeine and sodium bicarbonate was dependent on the type of task performed (e.g., endurance, power, etc.) [46]. For instance, Rezaei et al. [47] evaluated the effect of combined and isolated caffeine and sodium bicarbonate on a Karate-Specific Aerobic Test (KSAT) protocol, with the hypothesis that combined supplement intake would result in higher KSAT performance, but no differences were found between combined and isolated intake. In another study conducted among 10 elite male cyclists, the performance of the 3 km cycling time trial was examined. At the end of the study, it was observed that the performance of the 3 km cycling time trial of isolated caffeine, isolated sodium bicarbonate, and combined intake of caffeine and sodium bicarbonate increased compared to the placebo group [25]. While sodium bicarbonate affects especially aerobic performance, caffeine appears to be effective in increasing acute strength [48].

The main reason for this can be explained by an increase in calcium release from the sarcoplasmic reticulum, accompanied by an increase in motor unit recruitment promoted by caffeine [8]. Caffeine-induced greater calcium mobilization from the sarcoplasmic reticulum is also associated with increased sensitivity of myofibrils to calcium [49]. Sodium bicarbonate, on the other hand, exerts its ergogenic effects by acutely increasing blood bicarbonate, leading to a greater flow of hydrogen ions and lactate moving from active muscles into the blood circulation. In this manner, the intramuscular pH balance is more easily achieved. After sodium bicarbonate consumption, the acute increase in blood bicarbonate levels and the changes in pH gradient between intracellular and extracellular environments support hydrogen ion flow from the exercise muscle to the blood circulation. This provides intracellular pH regulation and fatigue reduction [35].

The synergistic effect of caffeine and sodium bicarbonate increases performance by delaying fatigue in the central nervous system. This performance enhancement mechanism can be explained by decreased extracellular potassium accumulation and increased extracellular buffering capacity [50]. However, in our study, no statistically significant differences have been found between the placebo group and the combined intake of caffeine and sodium bicarbonate. When examining the findings of the literature, it seems that isolated sodium bicarbonate shows positive effects on aerobic performance but not necessarily on very short, highly metabolic-demanding activities. Regarding caffeine supplementation, isolated consumption generally leads to performance improvements, but its combination with sodium bicarbonate reveals mixed results [24]. This is in line with the findings of our study.

### 4.1. Limitations

This study has several limitations. The sample consisted of 19 male university students, which may limit the generalization of the results to women, older adults, or elite athletes. The duration of the intervention was short-term, potentially missing the long-term effects of caffeine and sodium bicarbonate supplementation on strength performance. The study focused exclusively on the IMTP test, which may not represent the effects of these supplements on other types of exercise or sports performance, particularly those involving dynamic or aerobic activities. Individual variability in response to caffeine and sodium bicarbonate supplementation was not fully explored, and fixed doses of caffeine (6 mg/kg) and sodium bicarbonate (0.3 g/kg) were used, which may not be optimal for all individuals. The research assessed only the acute effects of supplementation 60 min before exercise without considering the potential benefits or drawbacks of chronic supplementation. The scope of this study was limited to the IMTP test, which can be considered as an external load, and physiological parameters such as intramuscular pH concentration or blood lactate concentration were excluded from the evaluation. Additionally, the study was conducted in a controlled laboratory setting without a dietary log about the participant’s nutrition, which may not accurately reflect real-world conditions where various external factors could influence performance.

### 4.2. Practical Applicability

The practical applications of this study on the effects of pre-exercise isolated and combined caffeine and sodium bicarbonate consumption on IMTP performance are multifaceted, especially for athletes and coaches aiming to optimize training and competition results. The findings suggest that consuming caffeine 60 min before exercise can enhance submaximal strength, making it a valuable ergogenic aid for activities requiring high-intensity, short-duration force production. This can be particularly beneficial for sports that involve explosive movements, such as weightlifting, sprinting, and certain field sports. Coaches and athletes may consider integrating caffeine supplementation into their pre-exercise routines to gain a competitive edge. However, the study also indicates that sodium bicarbonate, alone or in combination with caffeine, does not provide additional benefits for submaximal strength in the IMTP tests. Therefore, resources and efforts could be better allocated by focusing on caffeine supplementation rather than sodium bicarbonate. These insights can help design more effective nutritional strategies to maximize athletic performance while also guiding further research into targeted supplementation protocols or some physiologic parameters like the intramuscular pH concentration and at least the blood lactate concentration can be evaluated.

## 5. Conclusions

The present study demonstrates that caffeine supplementation can positively influence IMTP performance, establishing it as a valuable ergogenic aid for athletes engaged in strength-based activities. Conversely, sodium bicarbonate, whether consumed alone or in conjunction with caffeine, does not enhance IMTP performance. These findings suggest that athletes and coaches should consider incorporating caffeine into their pre-exercise routines to improve performance in tasks requiring short bursts of strength. Furthermore, the effectiveness of these substances can vary depending on whether exercise is aerobic or anaerobic, highlighting the need for tailored supplementation strategies based on the specific demands of the sport. Future research should explore the differential impacts of caffeine and sodium bicarbonate on various types of exercise to develop more precise guidelines for their use to improve athletic performance.

## Figures and Tables

**Table 1:** Descriptive characteristics of the participants.

Variables	Mean ± SD
Age (years)	23.6 ± 1.6
Height (cm)	171 ± 9.4
Body weight (kg)	64.3 ± 9.6
Fat (%)	20.5 ± 7.3
Body mass index (kg/m^2^)	21.7 ± 2.4

**Table 2:** Results of statistical analysis.

Variable	Con	Pla	Caf	NaHCO_3_	Caf + NaHCO_3_	η^2^	Power	*p*
Peak (N)	1091.00 ± 100.00	1122.00 ± 95.00	1224.00 ± 92.00	1222.00 ± 74.00	1152.00 ± 109.00	0.109	0.976	0.100
Relative (N/Kg)	16.60 ± 0.01	17.20 ± 0.01	18.80 ± 0.01 *^†^	18.50 ± 0.01	18.20 ± 0.01	0.197	0.989	0.003

(N): Newtons. (N/Kg): Newtons per kilogram. Con: control. Pla: placebo. Caf: caffeine. NaHCO_3_: sodium bicarbonate. Caf + NaHCO_3_: caffeine + sodium bicarbonate. *: difference in the control group. ^†^: difference in the placebo group.

## Data Availability

The data presented in this study are available on the website https://osf.io/r6uz2/ (27 December 2022) with identifier 10.17605/OSF.IO/R6UZ2.
